# Environmental performance of ordinary and new generation concrete structures—a comparative analysis

**DOI:** 10.1007/s11356-018-3804-2

**Published:** 2018-12-14

**Authors:** Daniel Wałach, Piotr Dybeł, Joanna Sagan, Magdalena Gicala

**Affiliations:** AGH University of Science and Technology, Krakow, Poland

**Keywords:** High-performance concrete, Self-compacting concrete, Environmental analysis, Concrete structure, Sustainable construction, Integrated life cycle design

## Abstract

The development of concrete technology results in a new generation of cement-based concrete such as high-performance concrete, self-compacting concrete and high-performance, self-compacting concrete. These concretes are characterised by better parameters not only in terms of strength and durability but also rheology of the mixtures. Obtaining such properties requires the adoption of a different composition and proportion of ingredients than ordinary concrete. The greater share of cement in these concretes causes an increase in the energy consumption and emissions (per unit of concrete volume) at the production stage. However, use of new generation concrete allows for a reduction of overall dimensions of a structural element, due to the increased strength parameters. Such a solution may finally result in lower consumption of resources and energy, as well as a decrease of gas emissions. The article presents the results of a comparative environmental analysis of ordinary and new generation concrete structures.

## Introduction

Sustainable development requires a balance between the social, environmental and economic aspects for economic processes. Continuous development of technologies (including construction materials) should fulfil the requirements of the market, trying to maintain the above-mentioned balance at the same time; therefore any implemented solutions must be evaluated and compared in respect of many different criteria including environmental (General Assembly of United Nations 2015).

New generation concretes, such as high-performance concrete (HPC) with compressive strength above 60 MPa, self-compacting concrete (SCC) and their resultant, high-performance, self-compacting concrete (HPSCC) are an alternative to ordinary concrete. To obtain such properties, it is necessary to adopt the specific composition and proportion of ingredients (including the greater consumption of cement), which ultimately leads to impression that new generation concretes are less environmentally friendly.

Previously conducted research in the field of environmental assessment of structural and material solutions including concrete (Marinkowić 2013; Shoubi et al. 2013) has focused on single assessment indicators and the scope of the evaluation was limited. The aim of these works was to assess new generation concrete in reference to ordinary concrete, with expanded scope of assessment and a set of environmental indicators, making the evaluation more comprehensive. Moreover, the article details parameters substantially influencing the result of the assessment. For the assumptions made, the limit of using new-generation concretes efficiency, which can be used by designers as a tool for structure integrated design, was determined. In the article, the directions of possible construction optimization were indicated and it was emphasized that the design approach change can be a crucial element in the implementation of EU targets (2020, 2030) by the construction industry.

The structure of the article is as follows: the “Literature review” section contains a review of the literature, the next part describes the tools and methods used for analysis (“Tools and methods” section) including output compositions of concrete mixtures of the assessed solutions (“Composition of concrete mixtures” section), selected environmental indicators and their weights (“Environmental impact indicators” section), and the used method of establishing eco-indicators (“Eco-indicator” section). The “Description of the analysed structure” section includes information on the type, geometry, burdens and climate conditions of the analysed structural elements that are the type of functional equivalent of a building. In the “Results and discussion” section, the results of environmental analyses of the selected structural and material solutions were collected and the most favourable solution regarding the analysed criteria and assumed weights was indicated. General conclusions of these analyses are presented in the “Conclusions” section.

## Literature review

During the last few decades, new generation concretes have appeared (Kumar and Kaushik 2003). They are characterised by significantly better properties regarding resistance and durability, but also rheology (Ouchi 2001; Kumar and Kaushik 2003; Jalal et al. 2015). The following concretes are considered to be of the new generation: high-performance concrete (HPC) with resistance to compression above 60 MPa, self-compacting concrete (SCC) and their resultant, high-performance, self-compacting concrete (HPSCC) (Ajdukiewicz and Radomski 2002; Giergiczny et al. 2002; Szwabowski and Gołaszewski 2010; Jalal et al. 2015). HPSCC is able to fill formworks without the need of mechanical compacting and it is characterised by high resistance to compression and high level of durability. To obtain such properties, it is necessary to adopt the specific composition and proportion of ingredients (Sabet et al. 2013). Compared to ordinary concrete (NSC), mixtures of HPSCC have a larger content of Portland cement, superplasticizer (SP) and reactive mineral additives, usually in the form of silica fume (SF) (The Self-Compacting Concrete European Project Group 2005; De Schutter et al. 2008; Gesoğlu et al. 2009; Shoubi et al. 2013; Le et al. 2015). Taking all concrete ingredients, cement production is the most energy-consuming and contributes significantly to the emission of carbon dioxide. Therefore, compared to the technology of ordinary concrete, the technology of new generation concrete seems to be less environmentally friendly. It needs to be mentioned, however, that high resistance of HPC and HPSCC enables a reduction of structural elements sections as well as reinforcements, which finally results in lower material consumption index of structures and impact on the environment. Therefore, in engineering practice, while seeking material and structural solutions, it is necessary to implement an integrated design approach to the whole life cycle of a construction facility. A corresponding technology should be assessed only regarding that aspect (Mora et al. 2011).

Nowadays, this approach is considered to be crucial for concrete construction (Marinkowić 2013). Therefore, research is carried out to investigate the impact of design strategies on environmental as well as economic efficiency (Rohden and Garcez 2018), and solutions favourable to creating an environmental profile are desirable. For this purpose, replacing a certain amount of cement with by-products of the industry processes, characterised by lower environmental loads than the original binder, is proposed (Shoubi et al. 2013). Although, research is usually limited to selected environmental impacts or aspects as well as life cycle stages of a building, there are also more comprehensive analyses taking into account the set of environmental indicators (Fiala et al. 2011) and focused on the comparative analysis of ordinary and high-performance concretes. Their results clearly show that by increasing the mechanical strength of concrete, the environmental impact per cubic meter of mixture increases due to the increase in cement content. However, it is possible to reduce the amount of concrete needed to build a given structural element (Habert et al. 2012).

## Tools and methods

The performed analysis consists of two parts in general. The first part includes the analysis of structure regarding meeting all output functional and usage requirements. The analysis was carried out according to standards (EN 1990; EN 1991; EN 1992; EN 206-1) ensuring ultimate and serviceability limit state. Autodesk Robot Structural Analysis Professional 2013 software was used for the calculations. It is based on the finite elements method; therefore it allowed for static calculations of particular structural elements, slabs and columns. Usage of different kinds of concrete, HPC and HPSCC, to particular structural elements, considering the permanent consumption of reinforcing steel in various variants, was analysed. The calculations helped determine geometrical dimensions of structural elements and consumption of particular kinds of concrete in the analysed solutions.

The second part of the analysis concerns the environmental assessment of the selected structural and material related solutions. For the purpose of the analysis, EPD (Environmental Product Declarations) of semi-products of concrete mixtures were used (EN 15804). By means of calculation of their proportion in a mixture, and then in a structural element, consumption of natural resources and emissions of the discussed variants were established. The performed analyses are of a systemic character. They apply to the stage of material production (modules A1–A3), transport (A4), as well as laying and compacting of the mixture at the site (A5) (EN 15643-1). Building Research Establishment (BRE 2005; Mundy 2015) guidelines were applied to standardise and determine the environmental impact of the selected results. Based on that, the analysed solutions were ordered in a hierarchical model.

### Composition of concrete mixtures

The study was performed for ordinary concrete (NSC), HPC and HPSCC mixtures in which the amount of silica fume was changed (0, 5, 10 and 15% of cement mass), while the binder content (500 kg/m^3^) and water-binder ratio (0.32) were constant. The composition of the mixtures was developed based on own experiences and work guidelines (Neville and Aitcin 1998; Ajdukiewicz and Radomski 2002; The Self-Compacting Concrete European Project Group 2005; Spak 2017). Two HPC mixtures were used, characterised by the same water-binder (w/b) ratio and components with the same properties as the HPSCC mixtures. The composition of the concrete mixtures (in kg per cubic meter) and obtained average values of compressive strength determined on cubic samples are given in Table 1. On the basis of the EN 206-1 standard, concretes were qualified for the following strength classes: NSC of C30/37 class, HPC1-2 of C70/85 class, HPSCC1-4 of C70/85 class.

**Table 1:** Composition of concrete mixtures [kg/m^3^]

Ingredients	NSC	HPC1	HPC2	HPSCC1	HPSCC2	HPSCC3	HPSCC4
Cement CEM I	380	500	455	500	476	455	435
Water	190	160	160	160	160	160	160
Sand 0/2 mm	580	668	668	840	840	840	840
Gravel 2/8 Gravel 8/16 Basalt aggregate 2/8 mm	400 860 –	– – 1240	– – 1240	– – 990	– – 990	– – 990	– – 990
Silica fume	–	–	45	–	24	45	65
Superplasticizer	3.8	3.25	4.05	5.55	5.85	6.15	7
Average compressive strength [MPa]	41.2	89.2	91.1	90.5	95.7	94.3	91.4
Coefficient of variation [%]	3.2	3.1	3.8	4.1	4.3	2.7	2.9

### Environmental impact indicators

On the basis of the studies of Abbe and Hamilton (2017), 11 environmental indicators were used for the purpose of assessment: global warming potential (GWP), net use of fresh water (FW), depletion potential of the stratospheric ozone layer (ODP), acidification potential of soil and water (AP), eutrophication potential (EP), radioactive waste disposed (RWD), abiotic depletion potential for non-fossil resources (ADPE), formation potential of tropospheric ozone (POCP), hazardous waste disposed (HWD), abiotic depletion potential for fossil resources (ADPF) and non-hazardous waste disposed (NHWD). At the stage of their selection, a reliable establishment of indicator values based on available databases and simultaneous analysis of the multiple results were taken into consideration. According to the authors, the remaining aspects defined in the (EN 15643-2) standard are essentially correlated with the above-mentioned set of indicators (BRE 2005). Therefore, limitation up to the mentioned 11 indicators is justified.

Concrete is a material formed from the mixing of particular ingredients, gaining its properties in the result of cement hydration. At the same time, its environmental profile is determined by the features of individual ingredients of a concrete mixture and the processes allowing its production, transport, laying and compacting. Table 2 presents the unit values of the environmental indicators of semi-products of concrete mixtures—the data comes from Environmental Product Declarations (EPD) (Danish Technological Institute 2013; DBC 2014; Holcim 2014; CEMBUREAU 2015; NCC Industry AB 2016). Since the silica fume is a by-product of industrial processes, its impact ought to be indicated by allocation procedure. However, the difference between the GWP indicator, in terms of economic allocation and non-allocation, is not significant (Van den Heede and De Belie 2012). Thus, only environmental impacts related to loading at power plant, depot and transport were assigned to silica fume.

**Table 2:** Unit values of environmental indicators of ingredients of the analysed concrete mixtures

	CEM I [1 t]	Water [1 kg]	Sand 0/2 [1 t]	Silica fume [1 t]	Gravel 2/8 crushed stone [1 t]	Gravel 2/8 and 8/16 natural [1 t]	Superplasticizer [1 kg]
GWP [kg Co_2_-eq.]	8.98E+02	5.70E-04	3.10E+00	3.92E+00	2.62E+00	3.10E+00	1.84E+00
FW [m^3^_eq_^.^]	9.50E+00	1.00E-03	1.17E+00	4.27E-01	5.91E-02	1.17E+00	5.70E-03
ODP [kg CFC11 eq.]	1.21E-07	2.35E-14	5.04E-10	9.88E-10	4.40E-11	5.04E-10	2.61E-10
AP [kg SO_2_ eq.]	1.48E+00	8.58E-07	4.33E-02	7.26E-03	2.05E-02	4.33E-02	2.38E-03
EP [kg (PO_4_)^3-^ eq.]	2.11E-01	2.48E-07	3.67E-03	1.05E-03	4.87E-03	3.67E-03	9.81E-04
RWD [kg]	1.00E-01	0.00E+00	0.00E+00	1.23E-04	1.68E-04	0.00E+00	7.24E-04
ADPE [kg Sb eq.]	1.10E-03	2.44E-10	2.11E-07	3.29E-07	8.34E-07	2.11E-07	1.11E-06
POCP [kg Ethene eq.]	1.42E-01	8.53E-08	6.64E-03	5.49E-04	2.49E-03	6.64E-03	2.47E-04
HWD [kg]	1.20E-01	0.00E+00	0.00E+00	0.00E+00	1.97E-06	0.00E+00	2.69E-03
ADPF [MJ]	3.44E+03	5.38E-03	3.99E+01	4.33E+01	3.08E+01	3.99E+01	2.79E+01
NHWD [kg]	1.50E+00	0.00E+00	0.00E+00	7.95E-02	8.95E-02	0.00E+00	7.78E-03

Environmental indicators for the processes of laying and compacting of concrete were calculated based on the energy consumption of the aforementioned processes (The International Federation for Structural Concrete 2004) (Table 3), using *PL:Electricity grid mix ts*. It should be noted that self-compacting concrete mixtures do not require mechanical vibration.

**Table 3:** Energy consumption of the processes of laying and compacting of concrete (The International Federation for Structural Concrete 2004)

Process	Type	Process energy consumption
Concrete pump	electricity	0.49 kWh/m^3^
Flexible stick-type vibrator	electricity	0.29 kWh/m^3^

Environmental indicators for the processes of transport were determined in GaBi software, using *EU-28:Transport*, *truck-trailer* (*40 t total cap*., *24.7 t payload*) *ts* and *EU-28: Transport*, *small truck* (*up to 14 t total cap., 9.3 t payload*) *ts.*, assuming transport at the distance of 100 km, capacity use indicator 85% and fuel consumption at the level of 1.7 l/100 t km. Adopted transport distance results from the assumption that at such a distance (borderline) transport of the mixture by concrete truck is more logistically effective than the implementation of the concrete mixing plant at the construction site.

### Eco-indicator

For the purpose of assessment of environmental impacts, the Ecopoint unit was used (Mundy 2015), where the standardization process is as follows (BRE 2005):

1 $$ {N}_i=\frac{S_i}{R_i} $$

where *i* is the categories of impact, *N*_*i*_standardized result, *S*_*i*_ characteristic value of environmental impact, and *R*_*i*_ reference value (annually per resident of EU-28).

Reference values for GWP, FW, ODP, AP, EP, ADPE, POCP, ADPF and NHWD were adopted on the basis of BRE (2005), Mundy (2015) and Aymard and Botta-Genoulaz (2016). For HWD, the reference value was determined based on statistics published by Eurostat (2014), and for RWD, according to Nuclear Energy Agency Organisation for Economic Co-Operation and Development (2010) (Table 4).

**Table 4:** Reference values (annually/per resident of EU-28)

GWP^*^	ODP	AP	EP	POCP	ADPE	ADPF	FW	HWD	NHWD	RWD
12,300	0.22	71.20	32.50	21.50	39.10	273,000	377	187.43	3750	3.91

*Units according to characteristic values of indicators

The weights of impact indicators come from surveys and expert interviews carried out in 2015 by Abbe and Hamilton (2017). Indicators and their weights used for assessment and comparison of variants are presented in Table 5.

**Table 5:** Environmental impact indicators and their weights

Criterion weight* [%]										
GWP	ODP	AP	EP	POCP	ADPE	ADPF	FW	HWD	NHWD	RWD
24.1	13.5	8.4	8.2	5.8	6.6	4.0	15.2	5.0	2.1	7.0

*According to Abbe and Hamilton (2017)

After standardization and weighing of the values of the selected environmental indicators, the value of Ecopoint (E_p_) was calculated according to the following formula:

2 $$ {E}_p={\sum}_{n=1}^i{N}_i\bullet {w}_i $$

where:*N*_*i*_ is the standardized result of environmental impact for ith category, *w*_*i*_ the weight of ith category.

## Description of the analysed structure

The structural and material analysis concerned a 12-floor office building, of the following dimensions—in a view of 50 × 25 m and 3.7 m height of an individual floor. The object is a column- and slab-based structure with two internal shanks.

The calculation model assumed the impact of forces coming from the deadweight of individual structural elements, exploitation and climate burdens to the building's structures. Combinations of these burdens were performed based on a standard (EN 1991). Computer simulation generated automatic combinations based on a method of partial ratios. The building’s foundation was not included in the analysis.

**Fig. 1:**
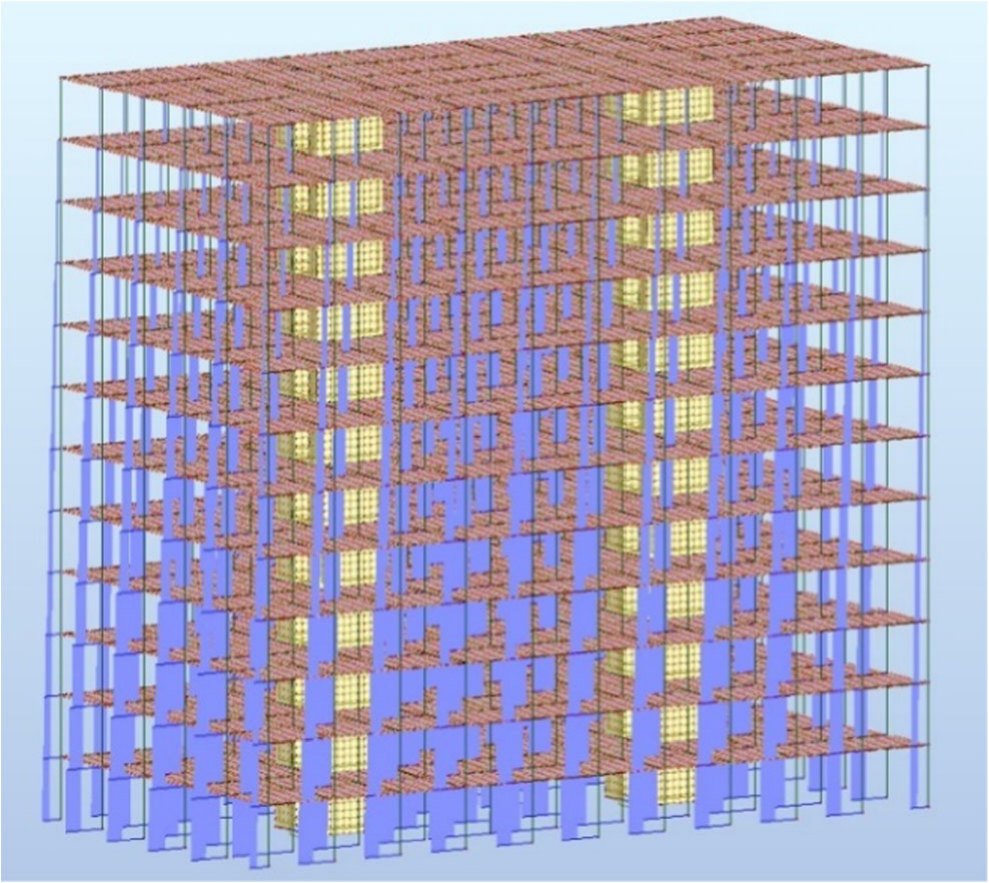
Envelope of axial forces in columns of the analysed model

**Fig. 2:**
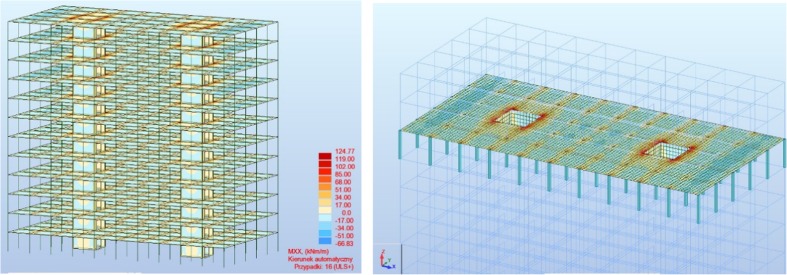
Map of Mxx moments (longitudinal direction) on slabs and shanks

Static calculations were applied to the determination of sectional forces values in individual structural elements (Figs. 1 and 2).

The structural design regarding ultimate and serviceability limit states was made in variants, assuming that the supporting structure of the building (slabs and columns) will be made of the following types of concrete:

- NSC of C30/37 class,
- HPC1-2 of C70/85 class,
- HPSCC1-4 of C70/85 class.

Use of high-class concrete in the analysed building allowed for a reduction of concrete amounts compared to ordinary concrete, in particular for the structural elements. This reduction mostly concerns the columns as, due to the character of their work, they eccentrically transfer compressing forces. Any significant reduction in thickness of slabs of higher class concrete was not observed. This results from the bidirectional work of slabs within their total area and the significant impact of bending moment on their load capacity. It should be noted that the calculations were made assuming constant reinforcement ratio in the particular cases.

Due to the different character of work of columns and slabs, Table 6 presents the results of the analysis of material consumption within particular elements and the whole structure.

**Table 6:** Results of concrete use in particular structural elements and the whole structure

	Whole structure [m^3^]	Columns [m^3^]	Slabs [m^3^]
NSC	2643.0	207.0	2436.0
HPC1-2 HPSCC1-4	2348.0	140.0	2208.0

## Results and discussion

### Environmental indicators of concrete mixtures

In the first stage, the standardized and weighed environmental indicators of the concrete mixtures were analysed. Studies prove that, in terms of assumed model parameters, the construction process of concrete building impacts worsens the ecosystem quality to the greatest extent by the phenomenon of global warming (59.18% t.i., total impact), use of freshwater (22.65% t.i.) and acidification (6.80% t.i.). A large total impact is also attributed to radioactive waste (5.99% t.i.) of naturally occurring radioactive materials (NORM), mainly coming from solid fuel burning in the process of energy production.

**Fig 3:**
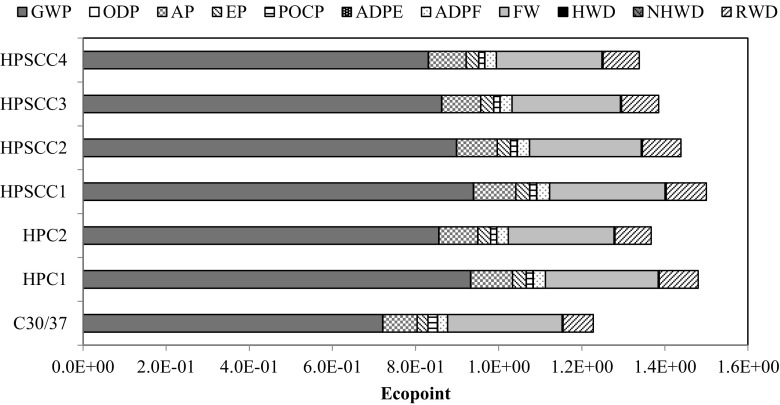
Environmental indicators of concrete mixtures per unit [m^3^]

Converting into a volume unit, and within the analysed modules of A1–A5, ordinary concrete (NSC) showed the least impact on the environment (Ep = 1.23) from all the analysed materials. Comparing the environmental impacts of the other variants, it is observed that with the decrease in the amount of cement in the concrete mixtures and the simultaneous increase in the environmentally friendly substitute material, the Ecopoint values decrease. In the case of HPSCC1-4 mixtures, it was found that replacing 10 kg of cement with silica fume reduces the Ecopoint value by approx. 1.7% (including the corresponding increased share of the superplasticizer) (Fig. 3).

**Fig 4:**
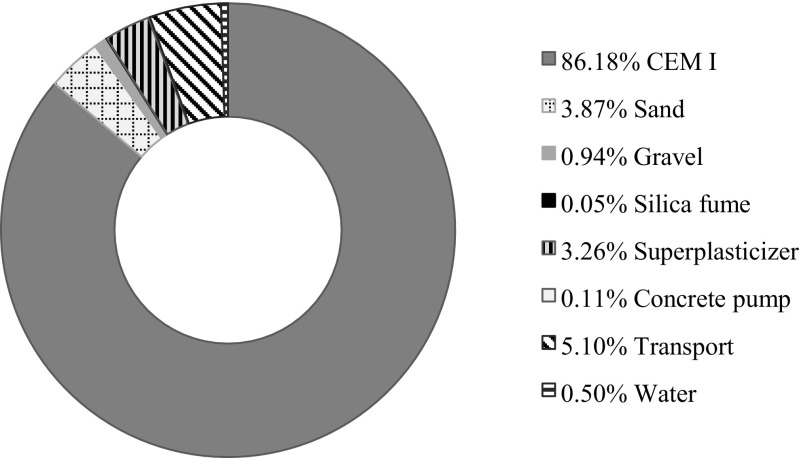
Participation of processes and semi-products of HPSCC4 concrete mixture in the structure’s total impact on the environment

The final result and advantage of the NSC over the other options is therefore closely related to the high proportion of cement in new generation concretes, whose production results in high emissivity, energy and water consumption. Its proportion in the total impact of HPSCC4 is significant and it equals 86.18% (Fig. 4). Statistics in the other mixtures are similar, also for NSC mixture. Among the other components of concrete mixture, sand and superplasticizer have significant influence on the environmental profile. An admixture that is a chemical substance does not remain environmentally neutral. The key influence of transport (over 5%) should be emphasized and in the case of significant impacts associated with the transport, its parameters should be carefully considered or other alternative solutions ought to be adopted.

**Fig. 5:**
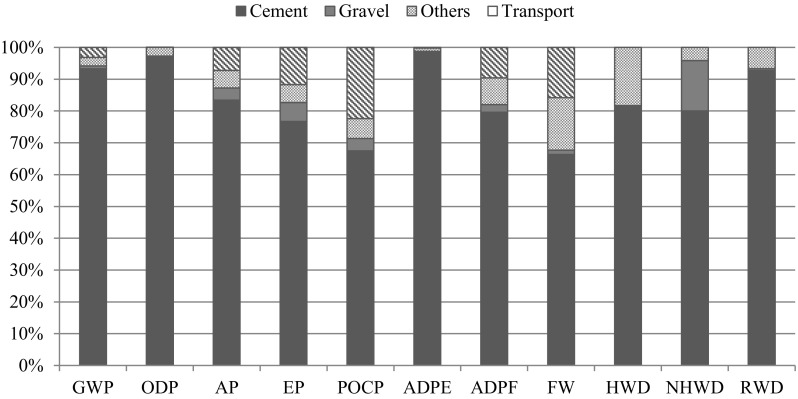
Participation of cement, gravel, transport and other components and processes in NSC environmental indicators

Considering the impact of cement, aggregates, transport process as well as a set of other components and processes related to concrete’s production, significant domination of binder, regarding the environmental profile of NSC, was observed (Fig. 5).

This trend was also found in (Marinkowić 2013)—comparative analysis is presented in the Table 7. Significant difference between the results was observed regarding POCP and EP. For these indicators, high contribution in environmental total impact has transport process. Its influence is caused particularly by the distance of transport and set of parameters such as engine’s class and type of road.

**Table 7:** Contribution of cement production to category indicator results

Indicator	Own results	Marinkowić (2013)
GWP	93.27%	93%
AP	83.40%	86%
EP	76.74%	81%
POCP	67.45%	83%

The Eco-indicator of high-performance concrete can be reduced by means of exchanging of some amount of cement through silica fume which also has binding properties (Shoubi et al. 2013). In HPSCC4, the amount of cement was reduced by 15%, which causes the decrease of the Eco-indicator by approximately 11% (compared to HPSCC1). The recipe of HPC mixtures is more complex and varied in quantity as well as quality of its components, thus environmental indicators per unit could be more divergent.

Considering the above, in order to reduce the negative impact of concrete structure on the environment in terms of both high-performance concrete and normal strength concrete, substitution of cement with alternative binders, such as silica fume, fly ash, should be taken into account. While the total EU production of concrete is 224 million m^3^ (ERMCO 2014), substitution of cement in a concrete mix can result in significant environmental savings. Replacing cement in a concrete mix with an alternative binder of 5, 10 to 20% reduces the total GHG (greenhouse gases) emissions in the EU by 0.06%, 0.12 to 0.25%, respectively, which is 11.3 million tonnes of CO_2_ equivalent in the last case. Further possibilities of reducing the environmental impact of concrete industry concern the energy sources (coal, nuclear, hydro) and the technology of cement production. However, one should be aware that direct emissions of cement come from chemical process called calcination, which involves the calcium carbonate breaking down into calcium oxide and CO_2_. This process accounts for 50% of all emissions from cement production.

Of equal importance for the environment is the issue of concrete mix and semi-finished products transport. Among the parameters influencing the environmental efficiency, the type of transport means and the transport distance should be mentioned. The transport distance of raw materials and semi-products is often related to the location of the rock deposit. In the case of large distances, an effective solution is partial substitution of natural crushed coarse aggregate with coarse recycled aggregate, limiting the transport distance of debris from locally demolished objects. According to (Mankelow et al. 2010), the average transport distance of a natural aggregate equals 40 km.

On the other hand, the transport distance of aggregates from concrete recycling (RCA) ranges from 10 to 30 km (Wittstock et al. 2012). Replacing natural aggregate with recycled aggregate may include 20% or more of total volume of aggregates in concrete mixtures (Gonçalves 2007). Considering that 224 million tonnes of aggregate is used in the EU for construction purposes (calculated based on the average proportion of aggregates in the concrete mix) and taking into account only the aggregate transport process (not to mention the profit from the recovery of concrete), it is estimated that the construction sector in the EU is able to reduce GHG emissions from 87,750 tonnes of CO_2_ eq. annually.

Further reductions of individual environmental indicators in terms of the analysed parameters are also possible. However, the above examples indicate the directions and scale of the reduction of concrete environmental impact while changing the design approach to preparing a concrete mix.

### Environmental indicators of elements and whole structure

**Fig. 6:**
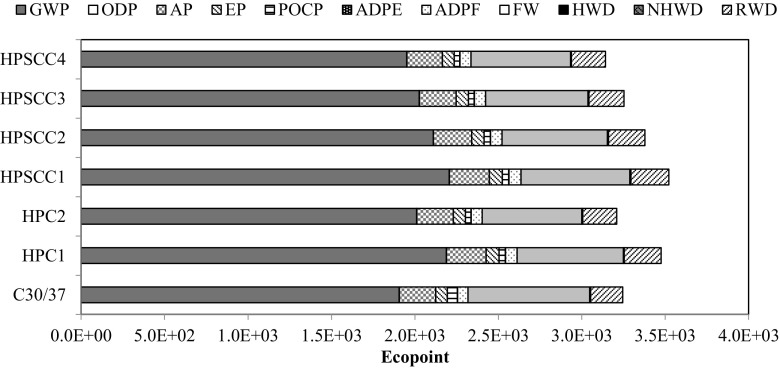
Environmental indicators of concrete mixtures per structure

Further, the values of environmental impact indicators of the assumed structure variants were compared and analysed (Fig. 6). Due to the higher class of new generation concrete, it was possible to reduce the dimensions of the structural elements (Table 6) by 11.16% in total. Considering the whole structure, the most advantageous solution, regarding levels of total environmental impact is alternatively using HPSCC4 (EP = 3142), subsequently HPC2 (EP = 3210) and then NSC (EP = 3245).

**Fig. 7:**
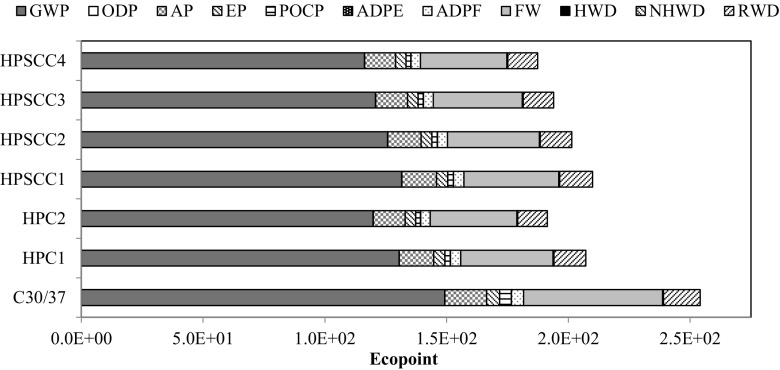
Environmental indicators for columns

**Fig. 8:**
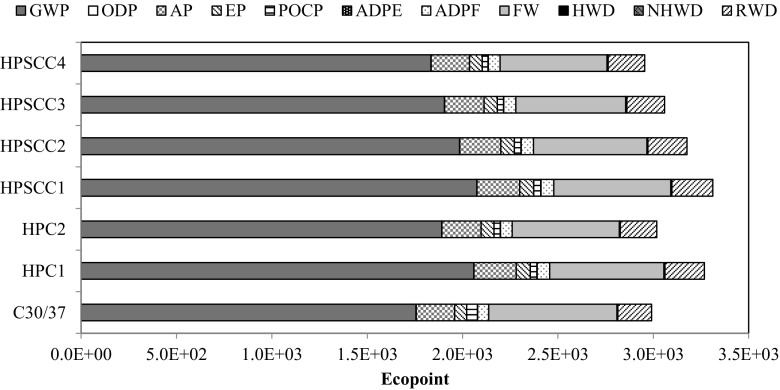
Environmental indicators for slabs

The last stage of analysis included calculation of total impact of main structural elements (columns and slabs). On this basis, material optimisation in the discussed structure was made regarding the environmental issue. The high resistance to compression of high-performance concrete allowed for a reduction of columns’ volume by 32.36%, and slabs’ volume only by 9.35%—this was caused by the character of the work of the structure. Its result was that columns made of HPSCC4 were the most favourable solution, and the use of any concrete of the new generation concrete group is more beneficial than the use of ordinary concrete (NSC) for these purposes (Fig. 7). However, in the case of slab-based structures (Fig. 8), only the use of self-compacting concrete, having 15% of silica fume in the cement mass, generated the result that is comparable to the use of ordinary concrete (Ep_HPSCC4_ = 2955; Ep_NSC_ = 2991).

Obtained results confirm the findings received by other researchers (Fiala et al. 2011; Habert et al. 2012), which provide results in narrowed set of indicators, such as GWP, EP, AP and ODP—due to the significant impact of this indicators which was highlighted (Smith 2003; Ramanathan and Feng 2009). Habert et al. (2012) indicate that environmental indicators on the structure level depend on its type, and can be lower for HPC by more than 20% (Table 8).

**Table 8:** Reduction of environmental indicators in HPSCC compared to NSC

Criterion	Own results^*^	Habert et al. (2012)
GWP	21.90%	20%
EP	20.52%	13%
AP	25.51%	16%
ODP	22.44%	10%

*Based on HPSCC4

**Fig. 9:**
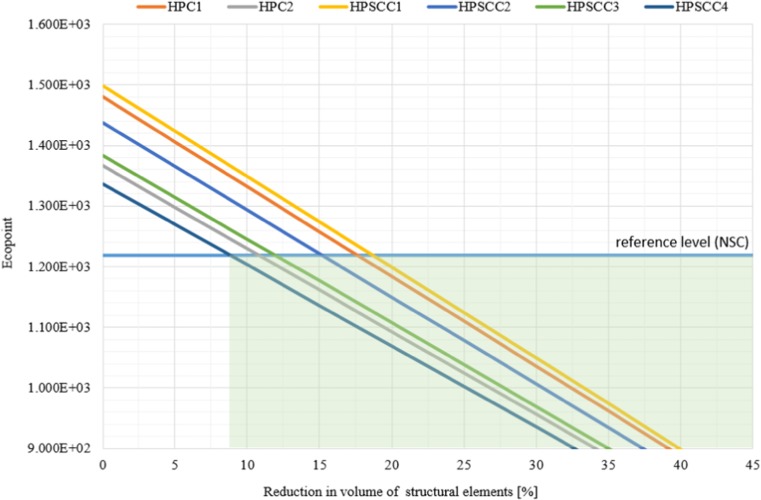
Limit (minimum level) of reduction of HPC structural elements volume compared to ordinary concrete (C30/37) structures (horizontal line)

Therefore, a general conclusion can be made stating that the level of reduction of construction material in a given element is a key parameter regarding the structure’s impact on the environment. Figure 9 presents the level of reduction of construction material for the use of new generation concrete as being most favourable to the environment compared to ordinary concrete (calculations per 1000 m^3^ of ordinary concrete). The analysis proves that HPSCC4 requires the lowest level of reduction (at 8.9%), while HPSCC1 needs the highest level at 18.7%, which is caused by the cement proportion in a mixture.

## Conclusions

The conducted studies included the environmental assessment of structural- and material-related solutions in the technologies of ordinary and high-performance concrete within the modules of A1–A5. The results prove the necessity to assess materials in building engineering regarding their widespread use. The values of indicators of new generation concrete total impact, calculated in a volume unit of concrete mix, can lead to erroneous conclusions on their negative impact on the environment. The use of new generation concrete of high resistance allows a reduction of total environmental impact of the structure by means of a reduction of the geometrical dimensions of the structure, what is particularly observed in compressed elements (columns) rather than bending elements (slabs). Compared to ordinary concrete, for the analysed high-performance concrete mixtures, a reduction of structural dimensions from 8.9 to 18.7% is required, depending on the amount of cement in the concrete mix. The high indicator of total impact of ordinary concrete on the environment is caused by the effect of global warming (58.18% t.i., total impact), use of freshwater (22.65% t.i.), acidification (6.80% t.i.) and emission of radioactive waste (5.99% t.i.). This is mainly caused by the processes of cement production. Participation of this semi-product in the ordinary concrete mixture on environment total impact equals 82.71%. Since the proportion of cement is higher in the high-performance concrete mixtures than in ordinary concrete, the reduction of cement amounts by means of use of silica fume provides additional opportunities to reduce the Eco-indicator (up to 11% in the studies).

Therefore, preparing different variants of materials and calculation of total impact of the structure based on the environmental profile of material, as well as characteristics of the work of the structure, is a favourable approach to structural design.

The presented approach and the obtained results may become a useful tool for designers implementing the postulate of integrated life cycle design of structures, detailed in The International Federation for Structural Concrete 2004. However, it should be emphasized that the results of the assessments depend on normalisation factors and weights, which can vary over time depending on the assessment scale and ecological problems occurring in the area under consideration.

The obtained results are therefore not timeless and universal, but the presented approach shows the possible direction of reducing the environmental impact of the construction sector, through the structural and material optimization, which should also lead to an economic and environmental compromise. The legal requirement to carry out these optimizations within the construction sector could become an element of the political strategy in pursuit of EU objectives for the coming years (e.g. *A 2030 climate and energy framework*).

## References

[CR1] Abbe O and Hamilton L (2017) BRE Global Environmental Weighting for Construction Products using Selected Parameters from EN 15804. https://www.bre.co.uk/filelibrary/Materials/Environmental-weightings-15804_final.pdf. Accessed 13 March 2017

[CR2] Ajdukiewicz A, Radomski W (2002). Trends in the Polish research on high-performance concrete. Cem Concr Compos.

[CR3] Aymard V and Botta-Genoulaz V (2016) Normalisation in life-cycle assessment: consequences of new European factors on decision-making. Proc 6^th^ Int Conf on Information Systems, Logistics and Supply-chain ILS Conference 2016. An Int J ed. Taylor&Francis

[CR4] BRE (2005) Green Guide to Specification Materials Industry Briefing Note 3b: Normalisation. http://www.bre.co.uk/greenguide/files/NormalisationBriefingDocumentFinal.pdf. Accessed: 7 July 2017

[CR5] CEMBUREAU The European Cement Association (2015) Environmental Product Declaration (EPD) according to EN 15804 and ISO 14025. Portland Cement (CEM I) produced in Europe. https://cembureau.eu/media/1254/6117_cembureau_epd_cem_2015-02-01.pdf. Accessed: 20 July 2017

[CR6] EN 206-1. Concrete - Part 1: Specification, Performance, Production and Conformity: 2000

[CR7] EN 1992. Design of concrete structures - Part 1-1: General Rules, and Rules for Buildings: 2008

[CR8] EN 1991. Actions on structures - Part 1-4: General actions, Wind actions: 2005

[CR9] EN 15643-1. Sustainability of Construction Works - Sustainability Assessment of Buildings - Part 1: General Framework: 2010

[CR10] EN 15643-2. Sustainability of Construction Works - Sustainability Assessment of Buildings - Part 2: Framework for the Assessment of Environmental Performance: 2011

[CR11] EN 15804. Sustainability of construction works - Environmental product declarations - Core rules for the product category of construction products: 2012

[CR12] EN 1991. Actions on structures - Part 1-3: General actions, Snow loads: 2003

[CR13] Danish Technological Institute (2013) Environmental Product Declaration. Fly Ash for Concrete, Asphalt and Cement Production

[CR14] DBC (Deutsche Bauchemie e.V.) (2014) Environmental Product Declaration as per ISO 14025 and EN 15804. Concrete admixtures - Plasticizer and superplasticizer. https://bauchemie.vci.de/wiki/DBC_Muster-EPDs/Documents/DBC-model-EPD_admixtures_Plasticizer_Superplasicizer_2014-01-08.pdf. Accessed: 17 Mai 2017

[CR15] De Schutter G, Bartos PJM, Domone P, Gibbs J (2008). Self-compacting concrete.

[CR16] EN 1990. Basis of structural design: 2002

[CR17] EN 1991. Actions on structures - Part 1-1 General actions Densities, self-weight, imposed loads for buildings: 2002

[CR18] ERMCO. (2014) Ready-Mixed Concrete Industry Statistics. Year 2013. no. July: 21. http://www.ermco.eu/documents/statistics/ermco-statistics-y-2013-final-version.pdf. Accessed 8 Aug 2018

[CR19] Eurostat (2014) Generation of waste by waste category, hazardousness and NACE Rev. 2 activitu. http://appsso.eurostat.ec.europa.eu. Accessed: 7 July 2017

[CR20] Fiala C, Kynčlová M, Hájek P (2011) Potentials for reduction of environmental impacts of construction using high performance concrete. fib Symposium 2011 in Prague: Concrete engineering for excellence and efficiency, Prague

[CR21] General Assembly of United Nations (2015) Transforming our world: the 2030 Agenda for Sustainable Development A/RES/70/1 New York. http://www.un.org/ga/search/view_doc.asp?symbol=A/RES/70/1&Lang=E. Accessed 1 August 2017

[CR22] Gesoğlu M, Guneyisi E, Ozbay E (2009). Properties of self-compacting concretes made with binary, ternary, and quaternary cementitious blends of fly ash, blast furnace slag, and silica fume. Constr Build Mater.

[CR23] Giergiczny Z, Małolepszy J, Szwabowski J and Śliwiński J (2002) Cements with mineral additives in new generation concrete technology Instytut Śląski Sp. z o.o. w Opolu, Opole (in Polish)

[CR24] Gonçalves P.C.M (2007) Concrete with Recycled Aggregates. Commented Analysis of Existing Legislation. Lisbona

[CR25] Habert G, Arribe D, Dehove T, Espinasse L, Le Roy R (2012). Reducing environmental impact by increasing the strength of concrete: quantification of the improvement to concrete bridges. J Clean Prod.

[CR26] Holcim (2014) EPD of Aggregates, Acording to ISO 14020:2000, IS0 14025:2006, ISO 14040:2006, ISO 14044:2006, ISO 21930:2007, EN 15804:2012, UN CPC 375:2013 Edition 1, Romania. http://www.holcim.ro/fileadmin/templates/RO/doc/SD_Reports/HoRo_EPD_Agg_final.pdf. Accessed: 17 July 2017

[CR27] Jalal M, Pouladkhan A, Harandi OF, Jafari D (2015). Comparative study on effects of Class F fly ash, nano silica and silica fume on properties of high performance self compacting concrete. Constr Build Mater.

[CR28] Kumar P, Kaushik S (2003). Some trends in the use of concrete. ICJ.

[CR29] Le HT, Müller M, Siewert K, Ludwig HM (2015). The mix design for self-compacting high performance concrete containing various mineral admixtures. Mater Des.

[CR30] Mankelow J, Oyo-Ita D, and Birkin M. (2010) Assessing the Carbon Footprint of Transporting Primary Aggregates. Extractive Industry Geology, 41–45.

[CR31] Marinkowić S (2013). Life cycle assessment (LCA) aspects of concrete. Eco-efficient concrete.

[CR32] Mora R, Bitsuamlak G, Horvat M (2011). Integrated life-cycle design of building enclosures. Build Environ.

[CR33] Mundy J (2015) The Green Guide Explained. BRE Centre for Sustainable Products. http://www.bre.co.uk/filelibrary/greenguide/PDF/The-Green-Guide-Explained_March2015.pdf. Accessed: 6 July 2017

[CR34] NCC Industry AB (2016) Environmental Product Declaration for aggregates from the stationary crushing plant Ramnaslätt, According to EN 15804:2012+A1:2013, ISO 14044 and ISO 14025

[CR35] Neville A, Aitcin PC (1998). High performance concrete - an overview. Mater Struct.

[CR36] Nuclear Energy Agency Organisation for Economic Co-Operation and Development (2010) Radioactive Waste in Perspective. https://www.oecd-nea.org/ndd/pubs/2010/6350-waste-perspective.pdf. Accessed 7 July 2017

[CR37] Ouchi M (2001) Self-compacting concrete: development, applications and key technologies. Proceedings of the 26^th^ Conference on Our World in Concrete & Structures 1:89–97

[CR38] Ramanathan V, Feng Y (2009) Air pollution, greenhouse gases and climate change: global and regional perspectives. Atmos Environ 43:37–50. doi: 10.1016/j.atmosenv.2008.09.063

[CR39] Rohden AB, Garcez MR (2018). Increasing the sustainability potential of a reinforced concrete building through design strategies: Case study. CSCM.

[CR40] Sabet FA, Libre NA, Shekarchi M (2013). Mechanical and durability properties of self consolidating high performance concrete incorporating natural zeolite, silica fume and fly ash. Constr Build Mater.

[CR41] Shoubi MV (2013). Assessment of the roles of various cement replacements in achieving the sustainable and high performance concrete. IJAET.

[CR42] Smith VH (2003). Eutrophication of freshwater and coastal marine ecosystems a global problem. Environ Sci Pollut Res.

[CR43] Spak M (2017). Preparation of High-Performance Concrete for Adjusting of Possibility of its Usage in Building Practice. Key Eng Mater.

[CR44] Szwabowski J, Gołaszewski J (2010). The technology of self-compacting concrete.

[CR45] The International Federation for Structural Concrete (2004) Environmental design. fib Bulletin No. 28 State-of-art report ISBN: 978-2-88394-068-0

[CR46] The Self-Compacting Concrete European Project Group (2005) The European guidelines for self-compacting concrete, specification, production and use p. 63. http://www.efnarc.org/pdf/SCCGuidelinesMay2005.pdf. Accessed 28 July 2017

[CR47] Van den Heede P, De Belie N (2012). Strength related global warming potential of fly ash (+ silica fume) concrete with(out) mass/economic allocation of the by-products' impact. Proceedings of International Symposium on LCA and Construction-Civil Engineering and Buildings. RILEM Proceedings.

[CR48] Wittstock B. et al. (2012) EeBGuide Guidance Document. Operational Guidance for Life Cycle Assessment Studies of the Energy-Efficient Buildings Initiative, 1–360.

